# Classification of atopic dermatitis phenotypes according to allergic sensitization by cluster analysis

**DOI:** 10.1016/j.waojou.2022.100671

**Published:** 2022-08-03

**Authors:** Hye Yung Yum, Ji Su Lee, Jung Min Bae, Sooyoung Lee, Yun Hee Kim, Myongsoon Sung, Song-I Yang, Jeongmin Lee, Mi-Hee Lee, Dong Hun Lee

**Affiliations:** a Department of Pediatrics, Seoul Medical Center, Seoul, South Korea; b Department of Dermatology, Seoul National University Hospital, Seoul, South Korea; c Department of Dermatology, College of Medicine, The Catholic University of Korea, Seoul, South Korea; d Department of Pediatrics, Ajou University School of Medicine, Suwon, South Korea; e Department of Pediatrics, Severance Hospital, Yonsei University College of Medicine, Seoul, South Korea; f Department of Pediatrics, Soonchunhyang University Gumi Hospital, Soonchunhyang University School of Medicine, Gumi, South Korea; g Department of Pediatrics, Hallym University Sacred Heart Hospital, Hallym University College of Medicine, Anyang, South Korea; h Department of Pediatrics, Yonsei University Wonju College of Medicine, Wonju, South Korea; i Department of Pediatrics, Incheon Medical Center, Incheon, South Korea

**Keywords:** Atopic dermatitis, Allergy, Sensitization, Children, Cluster analysis

## Abstract

A cluster study to classify atopic dermatitis (AD) phenotypes into subgroups is required to better understand and manage the disease owing to the heterogeneity of its clinical features. This study aimed to identify the phenotypic subgroups of childhood AD according to allergic sensitization. In 258 children with AD, hierarchical cluster analysis based on specific immunoglobulin (Ig) E sensitization revealed four distinct clusters. Cluster A (n = 71) revealed no IgE sensitization, whereas cluster B (n = 28) showed sensitization to egg white only. Cluster B was highly associated with early-onset AD (<3 months) and a family history of atopic diseases. Cluster C (n = 68) and D (n = 91), sensitized to multiple foods and inhalants, respectively, showed a higher prevalence of skin infection within the last 1 year than others. Cluster D was related to late-onset AD (>12 months) and had more atopic comorbidities. In addition, cluster D showed the most severely impaired health-related quality of life and more frequent use of immunosuppressants. Therefore, childhood AD can be classified into 4 clusters based on the allergic sensitization status, and clinical phenotypes and treatment strategy may be different according to clusters.

Dear Editor,

Atopic dermatitis (AD) is a common inflammatory skin disease that is frequently accompanied by other allergic diseases and substantially affects the quality of life (QoL) of patients and their families. AD manifests in a wide spectrum, ranging from very mild forms of dryness with minimal eczema to extremely severe forms of eczema.^1^^,^^2^ AD patients can be sensitive to diverse allergens, and allergic sensitization status is crucial in predicting the clinical course of AD.^3^ AD is a heterogeneous disease; hence, a cluster study to define subgroups that share similar phenotypes is warranted. Identifying subgroups can facilitate a better understanding of the underlying pathophysiology of AD, resulting in the selection of more effective treatments for individuals and prediction of disease prognosis.^2^ However, there have been only a few previous studies regarding differentiating phenotypes of AD, especially according to the allergic sensitization status. Furthermore, these studies involved only Caucasians or preschool children.^3^^,^^4^^,^^7^^,^^9^ Therefore, we performed a cluster analysis to identify phenotypic subgroups of Korean childhood AD according to allergic sensitization.

We enrolled 270 children under 18 years of age who were treated for AD at 7 secondary or tertiary hospitals between January 2017 and April 2018. AD was diagnosed by a medical specialist at the Department of Dermatology, Pediatric, or Internal Medicine according to the criteria of Hanifin and Rajka. Patients with severe skin diseases other than AD or systemic diseases other than allergies were excluded. Information regarding the clinical characteristics of AD was collected based on a structured questionnaire which was completed by the children or their parents. The data on serum total and allergen-specific immunoglobulin E (IgE) levels that were measured by multiple allergosorbent test chemiluminescent assay (MAST-CLA), ImmunoCAP (Thermo Fisher Scientific, Uppsala, Sweden), or skin prick test were retrospectively collected from electronic medical records. Allergen sensitization by MAST-CLA or ImmunoCAP was interpreted as positive when the result was class 1 or higher. Skin prick test was scored as positive when the wheal diameter was more than 2 mm greater than the negative control.^12^ The median duration between the date of questionnaire completion and the date of allergen sensitization test was 2 months (range, 0–33 months). This study was approved by the institutional review board.

Of the 270 children with AD, 258 children who had sufficient data on specific IgE sensitization were included in the cluster analysis. To identify the patterns of allergic sensitization in children with AD, we used an unsupervised agglomerative hierarchical clustering analysis with Ward's method and binary distance. Specific IgE sensitization status of 12 most common allergens (6 food and 6 inhalant allergens) in Korean children were used as variables: egg white, cow's milk, peanut, soybean, fish, wheat, *Dermatophagoides pteronyssinus, Dermatophagoides farinae*, cat, dog, fungus, and pollen (Supplementary Table 1). The number of cluster groups was determined by considering the clustering results and their clinical relevance. Differences in the clinical characteristics among different AD cluster groups were assessed using χ^2^ test or Fisher's exact test for categorical values and analysis of variance for continuous values. Post hoc analyses were conducted using Bonferroni correction when required. The results were presented as count (percentage) or mean ± standard deviation, and exceptions were marked separately. Statistical significance was set at *P* < 0.05. All statistical tests were performed using R (version 4.0.3, R Foundation for Statistical Computing, Vienna, Austria) software.

Of 258 patients, the mean age at enrollment was 3.9 years (±3.6 years), and the male-to-female ratio was 1.2:1. The majority (72.1%) of AD started before 1 year of age. The most common allergic comorbidity identified by a questionnaire was food allergy (50.4%), followed by allergic rhinitis (27.9%), allergic conjunctivitis (14.0%), asthma (7.8%), and chronic urticaria (5.4%). The median of health-related QoL (Children's Dermatology Life Quality Index) was 7 (range, 0–25). The average total IgE was 556 (±997.6) IU/ml. Regarding specific IgE sensitization, most of the patients (36.4%) were sensitized to three or more allergens, followed by 27.5% of patients without sensitization to any allergen. Approximately 60% of patients were sensitized to one or more food allergens. Among food allergens, egg white (47.7%), cow's milk (32.1%), and peanut (27.0%) were the most common. Inhalant sensitization (38.4%) was lower than food sensitization. Among inhalant allergens, *Dermatophagoides farinae* (33.9%) and *Dermatophagoides pteronyssinus* (32.4%) were the most common.

Hierarchical cluster analysis revealed four distinct clusters based on specific IgE sensitization (Fig. 1 and Table 1). Patients in cluster A (n = 71, called “intrinsic”) showed no IgE sensitization. Cluster A was less likely to have a food allergy (18.3%) than the other clusters (56.0%–72.1%). Patients in cluster B (n = 28, called “egg white only”) showed sensitization to egg white only. Patients in cluster B were younger than those in the other groups, and cluster B was significantly associated with early-onset AD (<3 months) (*P* < 0.001). In addition, cluster B revealed the highest prevalence of family history of allergic diseases (85.7%) despite no statistical significance. Patients in cluster C (n = 68, called “multiple foods”) exhibited sensitization to multiple foods, and patients in cluster D (n = 91, called “inhalants”) showed sensitization to inhalants. Patients in clusters C and D displayed significantly higher total IgE levels than those in the other clusters (*P* = 0.006). Although no sufficient statistical significance was noted, clusters C and D showed approximately twice a higher prevalence of skin infection in the last 1 year (20.6%–20.9%) than clusters A or B (10.7%–11.3%). The age of patients in cluster D was older than that in the other clusters. In addition, cluster D included late-onset AD (>12 months) (44.0%) more than the other clusters (7.1%–26.8%). In parallel, there were significantly more atopic comorbidities such as asthma, allergic rhinitis, and allergic conjunctivitis in cluster D than in other clusters (*P* = 0.006, *P* < 0.001, and *P* = 0.006 for asthma, allergic rhinitis, and allergic conjunctivitis, respectively). Cluster D revealed severely impaired health-related QoL than other clusters (P = 0.028). In addition, cluster D had a previous treatment history with systemic immunosuppressants (14.3%) more frequently than other clusters (0.0%–9.9%), although the difference was not statistically significant.

**Fig. 1:**
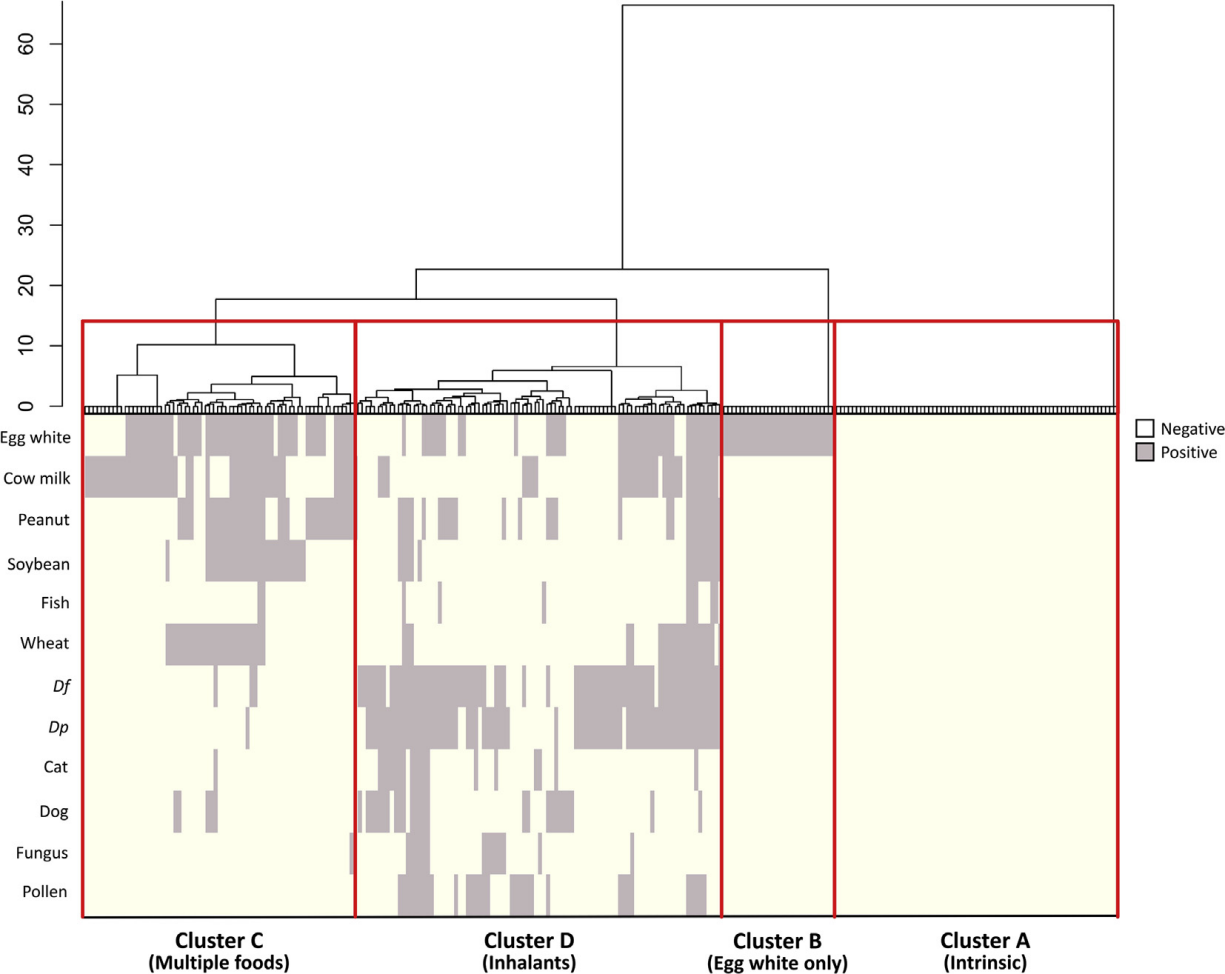
Results of hierarchical cluster analysis showing four distinct clusters of atopic dermatitis according to specific immunoglobulin E sensitization (*Df, Dermatophagoides farinae; Dp, Dermatophagoides pteronyssinus*).

**Table 1:** Clinical characteristics of 4 clusters of children with atopic dermatitis (AD) and comparison between clusters.

	A (Intrinsic)	B (Egg white only)	C (Multiple foods)	D (Inhalants)	*P* value (Cluster comparison)
Subject number, n (%)	71 (27.5%)	28 (10.8%)	68 (26.4%)	91 (35.3%)	
Sex ratio (M:F)	1.22:1 (39/32)	2.11:1 (19/9)	1.27:1 (38/30)	1.02:1 (46/45)	0.454
Age at enrollment (years), mean (± standard deviation)	3.3 (±3.4)	1.4 (±1.1).633 0322+6	2.3 (±1.7)	6.3 (±3.9)	<0.001
Age at AD onset, n (%)					<0.001
<3 months	25 (35.2%)	13 (46.4%)	21 (30.9%)	20 (22.0%)	
3–6 months	22 (31.0%)	11 (39.3%)	21 (30.9%)	20 (22.0%)	
6–12 months	5 (7.0%)	2 (7.1%)	15 (22.1%)	11 (12.1%)	
>12 months	19 (26.8%)	2 (7.1%)	11 (16.2%)	40 (44.0%)	
1st relative family history of allergic diseases, n (%)	46 (64.8%)	24 (85.7%)	47 (69.1%)	64 (70.3%)	0.236
Allergic comorbidities					
Asthma	4 (5.6%)	1 (3.6%)	1 (1.5%)	14 (15.4%)	0.006
Allergic rhinitis	12 (16.9%)	4 (14.3%)	17 (25.0%)	39 (42.9%)	<0.001
Allergic conjunctivitis	7 (9.9%)	2 (7.1%)	5 (7.4%)	22 (24.2%)	0.006
Chronic urticaria	5 (7.0%)	0 (0.0%)	4 (5.9%)	5 (5.5%)	0.907
Food allergy	13 (18.3%)	17 (60.7%)	49 (72.1%)	51 (56.0%)	<0.001
Health-related quality of life (0–30), median (range)	5.0 (0–20)	5.0 (0–24)	5.5 (0–23)	8.0 (0–25)	0.028
Total IgE (IU/ml), mean (± standard deviation)	213.2 (±627.7)	87.8 (±114.9)	763.0 (±1368.2)	694.7 (±891.2)	0.006
Previous treatment with systemic immunosuppressants	7 (9.9%)	0 (0.0%)	4 (5.9%)	13 (14.3%)	0.201
Skin infection in the last 1 year	8 (11.3%)	3 (10.7%)	14 (20.6%)	19 (20.9%)	0.260

Classically, AD is categorized into intrinsic (non-atopic) and extrinsic (atopic) types.^13^ The former is composed of AD with normal/low levels of total and specific serum IgE, whereas the latter is composed of AD with high levels of serum IgE. However, this dichotomized classification is extremely simple to represent heterogeneous phenotypes of AD.^2^^,^^4^ Hence, there have been discussions on classifying AD phenotypes by various factors including clinical features (onset age, severity, and disease course) and biomarkers (blood eosinophils and total/specific serum IgE).^2^, ^3^, ^4^, ^5^, ^6^, ^7^, ^8^, ^9^, ^10^, ^11^ In the present study, AD was classified into four distinct subgroups according to allergic sensitization status: cluster A (intrinsic), cluster B (egg white only), cluster C (multiple foods), and cluster D (inhalants). Allergen sensitization does not mean allergy to foods or inhalants. In 2019, Seo et al investigated AD phenotypes in children <3 years of age and classified AD into four subgroups: “early-onset, non-allergic AD”, “early-onset AD with high eosinophil and food sensitization”, “early-onset AD with high C-reactive protein”, and “middle-onset AD with inhalant sensitization”.^4^ They found that non-allergic AD showed the lowest severity, whereas AD with food sensitization showed the highest severity. In line with this finding, in our study, cluster A (intrinsic) had the lowest proportion of food allergies and other allergic diseases, less impaired health-related QoL, and low frequency of previous skin infection in the last year. In contrast to Seo et al, we divided AD children with food sensitization into 2 groups: cluster B (egg white only) and cluster C (multiple foods). Cluster B was significantly associated with early-onset (<3 months) AD and was less likely to have previous systemic immunosuppressant treatments or skin infection than cluster C. In parallel, Dharma et al demonstrated that children who were mono-sensitized to one food allergen at 1 year tended to have transient sensitization that disappeared in 3 years.^3^ Moreover, they showed that children with persistent poly-sensitization to foods are highly likely to develop allergic diseases.^3^ Considering the findings of our study and the previous study, AD children sensitized to egg white only may undergo a favorable clinical course, which should be differentiated from AD children sensitized to multiple food allergens. According to previous prospective cohorts, children are usually mono-sensitized to food before 1 year, whereas inhalant sensitization becomes more frequent with a concurrent decline in food sensitization after 1 year.^3^ In parallel, cluster D (inhalants) was associated with late-onset (>12 months) AD compared to other clusters in our study. Cluster D was prone to have allergic comorbidities such as asthma, allergic rhinitis, and allergic conjunctivitis than other clusters, but not food allergies. Cluster D seemed to have the worst clinical course: they were characterized by the most severely impaired health-related QoL and more frequently had previous treatment with systemic immunosuppressants and skin infection history. Our findings are compatible with those of previous studies that demonstrated that inhalant sensitization in AD is significantly associated with the severity of AD and increases the risk of developing other allergic diseases.^14^^,^^15^ Therefore, AD children with inhalant sensitization may require the most clinical attention to avoid disease flares and reduce later development of allergic diseases.

The present study had several limitations. First, we did not track temporal changes in allergic sensitization status over time. Because the sensitization status is unstable, especially in early life, the consideration of temporal changes in sensitization is required.^2^ Second, the data on clinical features of AD were collected based on a questionnaire that was completed by the children or their parents. There may be some errors due to memory distortion or the fact that they are not medical specialists. Third, this study included only Korean children with AD. Since AD phenotypes may differ among ethnicities, extrapolation of our findings to populations of other ethnicities may not be appropriate.^16^ Fourth, several methods to detect allergen sensitization were employed, because the allergen sensitization data were retrospectively collected from seven hospitals. Moreover, allergen sensitization, but not allergy, was tested and analyzed. Lastly, age might be a confounding factor because the age distribution was different among clusters. In the present study, most patients were preschool children or younger, but further studies validating these clusters in specific age groups are warranted. Despite several limitations, the present study still has a strength that it is a large study identifying childhood AD phenotypes according to allergic sensitization, which involved a broad range of age groups of Asian AD children.

In conclusion, childhood AD can be classified into four groups according to the allergic sensitization status. Children with AD without allergic sensitization or sensitization to egg white are expected to have a favorable clinical course. Meanwhile, AD children with inhalant sensitization have a high risk of uncontrolled disease and later development of other atopic diseases. Consequently, considerable clinical attention is required to control the disease in children with AD with inhalant sensitization.

## Abbreviations

AD, atopic dermatitis; Ig, immunoglobulin; QoL, quality of life; MAST-CLA, multiple allergosorbent test chemiluminescent assay.

## Funding source

Korean Academy of Asthma, Allergy and Clinical Immunology (KAAACI).

## Availability of data and materials

Data and materials are available from the corresponding author on reasonable request.

## Author contributions

1. The conception and design of the study, or acquisition of data, or analysis and interpretation of data: Hye Yung Yum, Ji Su Lee, Hye Yung Yum, Ji Su Lee, Jung Min Bae, Sooyoung Lee, Yun Hee Kim, Myongsoon Sung, Song-I Yang, Jeongmin Lee, Mi Hee Lee, Dong Hun Lee.

2. Drafting the article or revising it critically for important intellectual content: Hye Yung Yum, Ji Su Lee, Dong Hun Lee.

3. Final approval of the version to be submitted: Hye Yung Yum, Ji Su Lee, Hye Yung Yum, Ji Su Lee, Jung Min Bae, Sooyoung Lee, Yun Hee Kim, Myongsoon Sung, Song-I Yang, Jeongmin Lee, Mi Hee Lee, Dong Hun Lee.

## Authors’ consent for publication

All authors consented for publication.

## Declaration of competing interest

None declared.

## IRB approval status

Reviewed and approved by the Institutional Review Board of Seoul Medical Center (No. 2016-072).
