# Supplementary material for: Classification of atopic dermatitis phenotypes according to allergic sensitization by cluster analysis
Source: World Allergy Organ J. 2022 Aug 3;15(8):100671. doi: 10.1016/j.waojou.2022.100671 (PMC9357948; doi:10.1016/j.waojou.2022.100671)
Supplement: Multimedia component 1 [file mmc1.doc]

**Supplementary Table 1. Details of fungus and pollen allergens**

| **Fungus** | | **Pollen** | |
| --- | --- | --- | --- |
| MAST-CLA | ImmunoCAP | MAST-CLA | ImmunoCAP |
| *Aspergillus fumigatus*  *Alternaria alternata Candida albicans*  *Cladosporium herbarum*  *Penicillium notatum* | *Aspergillus fumigatus*  *Alternaria alternata* | sweet vernal grass  bermuda grass  orchard grass  timothy grass  reed grass  redtop  ryegrass  alder  birch  hazel  oak white  sycamore  sallow willow  poplar  ash  pine  Japanese cedar  acacia,  cypress  ragweed  mugwort  oxeye daisy  dandelion  English plantain, Russian thistle goldenrod  pigweed  Japanese hop | Grass mixture  Tree mixture  Weed mixture  Japanese hop |
